# Implementing Machine Learning in Interventional Cardiology: The Benefits Are Worth the Trouble

**DOI:** 10.3389/fcvm.2021.711401

**Published:** 2021-12-08

**Authors:** Walid Ben Ali, Ahmad Pesaranghader, Robert Avram, Pavel Overtchouk, Nils Perrin, Stéphane Laffite, Raymond Cartier, Reda Ibrahim, Thomas Modine, Julie G. Hussin

**Affiliations:** ^1^Service Médico-Chirurgical, Valvulopathies-Chirurgie Cardiaque-Cardiologie Interventionelle Structurelle, Hôpital Cardiologique de Haut Lévèque, Bordeaux, France; ^2^Structural Heart Program and Interventional Cardiology, Université de Montréal, Montreal Heart Institute, Montréal, QC, Canada; ^3^Faculty of Medicine, Research Center, Montreal Heart Institute, Université de Montréal, Montréal, QC, Canada; ^4^Computer Science and Operations Research Department, Mila (Quebec Artificial Intelligence Institute), Montreal, QC, Canada; ^5^Interventional Cardiology and Cardiovascular Surgery Centre Hospitalier Regional Universitaire de Lille (CHRU de Lille), Lille, France

**Keywords:** deep learning, interventional cardiology, cardiology, neural networks, prognosis

## Abstract

Driven by recent innovations and technological progress, the increasing quality and amount of biomedical data coupled with the advances in computing power allowed for much progress in artificial intelligence (AI) approaches for health and biomedical research. In interventional cardiology, the hope is for AI to provide automated analysis and deeper interpretation of data from electrocardiography, computed tomography, magnetic resonance imaging, and electronic health records, among others. Furthermore, high-performance predictive models supporting decision-making hold the potential to improve safety, diagnostic and prognostic prediction in patients undergoing interventional cardiology procedures. These applications include robotic-assisted percutaneous coronary intervention procedures and automatic assessment of coronary stenosis during diagnostic coronary angiograms. Machine learning (ML) has been used in these innovations that have improved the field of interventional cardiology, and more recently, deep Learning (DL) has emerged as one of the most successful branches of ML in many applications. It remains to be seen if DL approaches will have a major impact on current and future practice. DL-based predictive systems also have several limitations, including lack of interpretability and lack of generalizability due to cohort heterogeneity and low sample sizes. There are also challenges for the clinical implementation of these systems, such as ethical limits and data privacy. This review is intended to bring the attention of health practitioners and interventional cardiologists to the broad and helpful applications of ML and DL algorithms to date in the field. Their implementation challenges in daily practice and future applications in the field of interventional cardiology are also discussed.

## Introduction

In recent years, the field of interventional cardiology has been characterized by innovation and technological progress as clinicians, in partnership with specialists in molecular biology, biomedical engineering, biophysics and imaging technology, have raised interventional cardiology to a vibrant and dynamic subspecialty in mainstream medical practice. As this field matures, the range of opportunities and applications continues to broaden, and there is an increasing need to focus not only on the effectiveness of treatments but also on safety issues. Novel advancements in the field of artificial intelligence (AI) can facilitate, accelerate, and improve this ongoing progress.

Fluoroscopy has been for long the pillar of interventional cardiology, and recent technological advances shake interventionists' habits by proposing multiple novel solutions to the setbacks of the X ray-based 2-dimensional fluoroscopy imaging. Human-controlled assistant robots and cardiovascular image processing are technological advancements applied to catheterization laboratories and hybrid rooms (1–4). Additionally, among a large number of percutaneous coronary intervention (PCI) operators worldwide, there exists an experiential learning curve for procedural success as it's been shown that the adjusted risk of in-hospital mortality has been higher for PCI procedures performed by low- and intermediate-volume operators compared with those performed by high-volume operators (5, 6). While an operator's success probability can be formulated as a statistical problem itself, deep learning assisted augmented reality could help with improving the learning curve associated with operator PCI success. Although autonomous and semi-autonomous robots used in interventional cardiology are probably still a few years of development and universal deployment away from routine clinical use, the vision of the operating room of the future, implementing decision-support algorithms for procedure planning and operator guidance, progressively takes shape (7). In structural heart procedures and interventional cardiology this is in particular of significant importance as, for example, studies have shown that robotic-assisted PCI (R-PCI) compared with manual PCI reduces radiation exposure to the cath lab staff, which could also improve precision (8, 9). The concept of “surgical data science” has recently been proposed, a data-driven surgical healthcare approach enhanced by decision support algorithms, context-aware automated assistants, and improvement of surgical training by digital assistance (10). As cardiac disease treatment tends to be transferred from operating theaters to hybrid rooms and catheterization laboratories, such concepts could be adapted to the cardiac interventional community.

The ability to effectively extract and store health data, powered by increasing computation power and the ability to efficiently process it yielded an explosion of AI applications aiming at improving care and reducing costs (11). More recently, deep learning (DL) has emerged as one of the most successful branches of machine learning (ML) and artificial intelligence and implements diverse architectures of deeper neural networks (DNN) (12). Additionally to electrocardiogram (ECG) data and image/video processing, automated electronic health records (EHR), biological or genetic data mining to yield prognostic estimation of the probability of adverse outcomes, mortality included, have also been proposed for cardiology and general healthcare (13–17). And, there are signs that the implementation of AI into the catheterization laboratory has already started. For example, modeling in real-time the coronary fractional flow reverse (FFR) values from CT-angiography of the coronary vasculature using AI (instead of invasively using the dedicated wire) is feasible and if applied to coronary angiographies, it could accelerate the procedure, to reduce irradiation and to avoid possible complications associated with the wire (18, 19).

Despite the notable improvements in medical care that can be achieved using cutting edge analytical methods and algorithms in image and video processing, clinical decision support, robotic assistance, and advanced clinical database analysis, the current state of AI in interventional cardiology is in its very infancy. Yet, if practitioners and cardiologists in the field are aware and open to embracing these changes positively, it can foreseeably revolutionize interventional cardiology practice in the near future. Drawing the attention of researchers and practitioners in the field to this opportunity is the aim of this review. We first provide an overview of machine learning applications in interventional cardiology; subsequently, we discuss the demand for future improvements considering machine learning implementation challenges in daily practice and future applications in the field of interventional cardiology.

## Machine and Deep Learning Overview

In contrast to traditional static rule-based AI systems which are equipped with algorithms developed based on fact sheets and documented and approved clinical research subsequently validated to produce expected results, data-driven AI utilizes large datasets and complex statistical methodologies to discover new relationships between inputs, actions, and outcomes. These systems are not explicitly programmed to provide pre-determined outputs, but are heuristic, with the ability to learn and make judgements to yield improved decision making with minimal human intervention. Even though there is a large overlap between statistical modeling and ML techniques, a common understanding is that statistical models mainly refer to analysis and reporting over data, while ML is more concerned with prediction by being able to exploit and possibly improve data representation for the task of interest. In general, ML models developed for data-driven AI systems can be categorized into supervised, unsupervised, semi-supervised, or reinforcement learning (Table 1).

**Table 1:** Type of learning methods.

**Method**	**Mechanism**	**Implementation**
Supervised 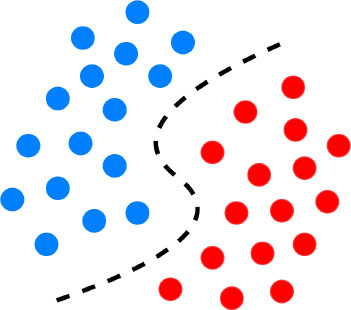	Uses labeled outcome data. The labels are typically assigned by experts in the field prior to model training (20, 21).	Involves tasks such as regression, classification, predictive modeling, survival analysis (22, 23).
Unsupervised 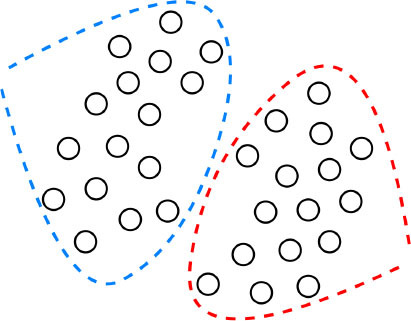	No labeled outcome data. We observe similarities, relationships, and if possible causality among groups and variables (20, 21).	Used for tasks such as dimensionality reduction, clustering, feature extraction (24).
Semi-supervised 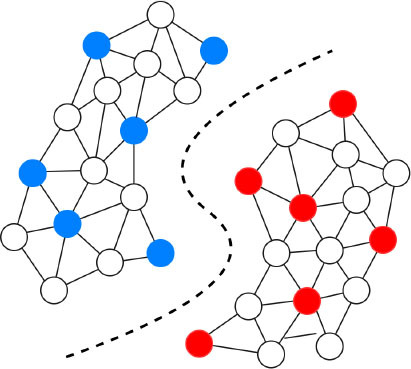	The input data contains both labeled and unlabeled outcome data (20, 21).	Labeled data is used to identify specific groups in data and their parameters. These data are then inputted to the algorithm along unlabeled data to explore the boundaries of the parameters (22, 23).
Reinforcement 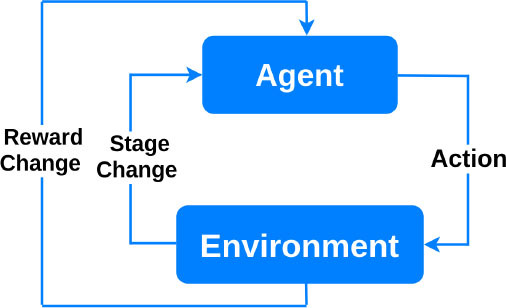	Based on behavioral psychology. The learning agent interacts with the environment to maximize a reward, and updates its parameters based on the feedback it receives from the choices it makes. The learning stops when the “reward” criteria are met to handle a decision-making function (25).	Can be used in medical imaging analytics and personalized prescription selection. Popular in automated robotics (26).

Supervised machine learning uses the independent features or variables to align and predict the known numerical or categorical validated outcome in the training dataset. Once properly trained, these models can then be used to predict outcomes when evaluating out-of-training samples (e.g., live patient cases). In the cardiovascular research, for example, supervised learning algorithms can identify and predict patterns in massive quantities of records, which are usually labeled by experts, and indicate the presence or absence of decreased systolic function on an echocardiogram or atrial fibrillation (AF) on an ECG (27). Regarding the model training, appropriate data preprocessing is typically done prior to separating data into distinct partitions of training, validation, and testing. This separation ensures fair and scientific evaluation and implementation of the model; while the validation partition would be employed for hyper-parameter selection of the model (e.g., numbers of layers in a DL network or how long model training should go on), test data must be used for final result reporting only.

Unsupervised learning, on the other hand, analyzes large amounts of typically unlabeled samples (e.g., EHR) to discover hidden patterns or innate structure which govern the existence of that data in order to substantially improve experts' understanding of that data including their involved representing features (28). In cardiology, for example, it has been shown that advanced unsupervised models such as causal networks can evaluate causal relationships among variables beyond partial correlations and thus play a fundamental step in risk prediction of cardiovascular disease (CVD) (29).

**Figure 1:**
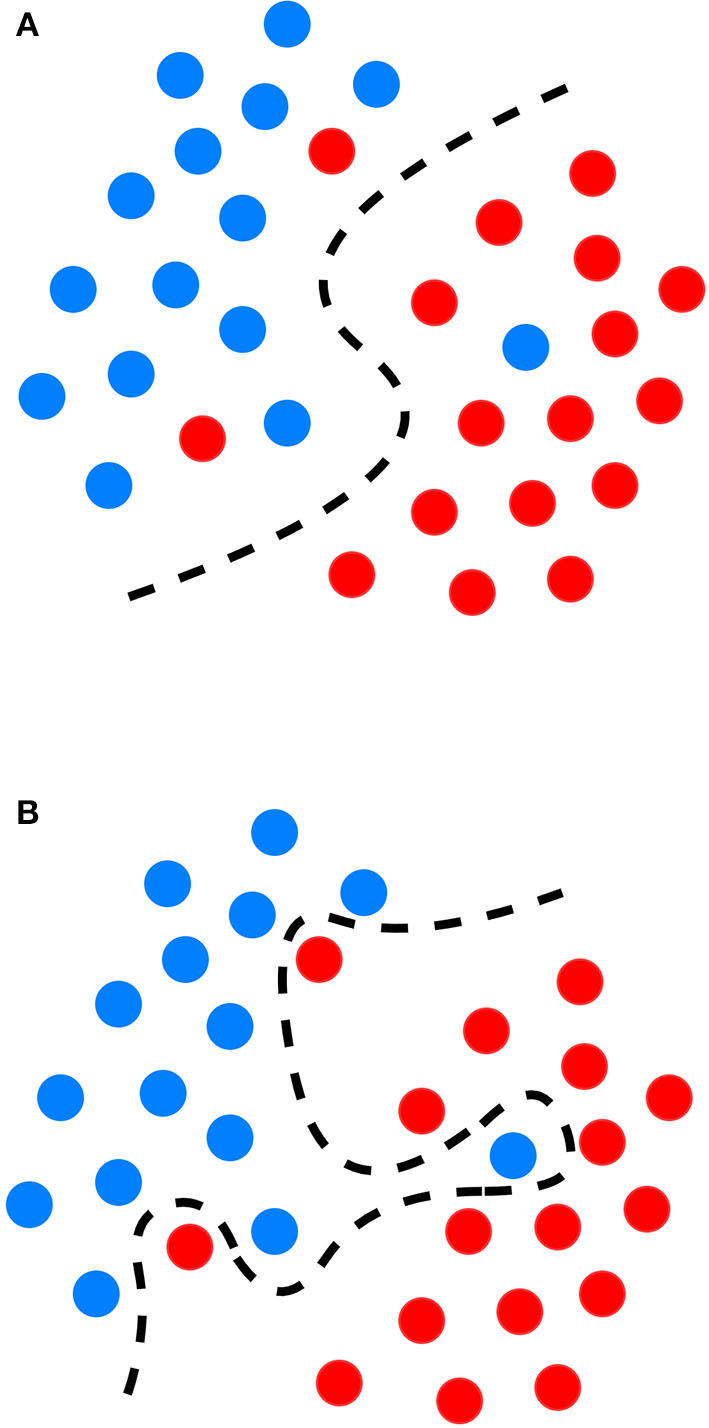
Graphical representation of the decision boundary (red line) in an optimal fitting **(A)** and overfitting model **(B)** overfitting describes a state of model which has poor generalizability due to excessive fitting of noise data presented in a training dataset.

Semi-supervised models work with datasets that are partially labeled. The labeling process of the unlabeled portion is done with the available training portion or with the help of unsupervised methods to do clustering first and then assign labels based on the characteristics of the recognized clusters (22). Generally, overfitting occurs when a supervised ML model approximates the system by available data correctly (Figure 1), but it is not able to produce proper results for verification or test data. It is especially a major problem in tasks for which enough labeled data is not available. Hence, semi-supervised learning can be a very useful technique for (semi-)automatically annotating lots of cases, e.g., to create a gold standard outcome label for all patients, without which it could be very expensive (22). Closely related to semi-supervised learning, transfer learning is also another strategy to address overfitting. It is an ML technique where a model developed for one task is reused as the starting point for a model on a second task. For proper transfer learning, however, the studied tasks should be conceptually related [e.g., catheter segmentation in X-ray fluoroscopy using synthetic data (30)].

Finally, *reinforcement learning* algorithms aim at maximizing a “reward” function (26). Reinforcement learning algorithms consist of an agent at a particular time interacting with an environment. An action is selected for each time point according to some selection policy. Transitions to the next state are then performed, and a reward is received depending on the result of the transition. The restricted learning model aims to maximize the expectation of long-term rewards from each state visited. In interventional cardiology, reinforcement learning can provide tools to optimize sequences of decisions for long-term outcomes such as improving ST-segment elevation myocardial infarction outcomes or reducing errors in ECG diagnosis. Optimization of treatment policies, real-time decisions and robot navigation are some other applications of reinforcement learning (31, 32).

Deep learning is applicable to any of the above-mentioned ML categories. It refers to the use of deep artificial neural networks to perform learning tasks. These networks are specific types of ML models where the learning happens in successive layers in such a way that each layer adds to the knowledge of the previous layer (33). DL models are capable of selecting and representing the right features on their own, thus eliminating the need for human intervention for manual definition of classification rules. For example, instead of defining that a ST elevation of ≥1 mm corresponds to a STEMI, DL models could automatically identify that the ST segment is the important feature, without any human input, and use that to predict the STEMI diagnosis. This revolutionary advancement in learning algorithms not only saves human time and labor but also minimizes the possibility of decision errors. For example, DL provided considerable advances in computer vision, a subfield in ML that matured first around 2012 and became highly popular in health and medicine, as they provide computers with the ability to learn visual features automatically from image or video content to produce diagnostic and prognostic information (34). It allowed automated analysis and interpretation of images such as computed tomography (CT), magnetic resonance imaging (MRI), electrocardiogram and echocardiography (35–38).

For instance, assessment of coronary stenosis during diagnostic coronary angiograms, one of the most commonly performed interventional cardiology procedures worldwide, is typically done using visual assessment. Thus, this method suffers from high inter-observer variability, operator bias and poor reproducibility (39–43). This variability in stenosis assessment has significant clinical implications, and likely contributes to inappropriate use of coronary artery bypass surgery in 17% of patients and of stents in 10% patients (40). While quantitative coronary angiography (QCA) using projection is able to validated quantitative measurements in coronary angiograms (44, 45), and is accepted as a gold standard for stenosis assessment, a study assessing 10 different QCA systems against a phantom stenosis gold-standard found absolute percentage differences of −26% to +29% in coronary stenosis assessments between systems and are semi-automatic, as they allow vessel contour modification by the human expert, which can bias the results. Deep learning algorithms can currently perform all tasks required for automatic interpretation of coronary angiograms, such as identification of left/right coronary arteries, anatomy description, vessel segmentation, stenosis localization and stenosis severity prediction leading to reduced variability and higher standardization of diagnostic angiograms (46, 47).

## Machine and Deep Learning for Cardiovascular Applications

Rather than a comprehensive review of all studies at the intersection of interventional cardiology and AI, this section aims at giving practitioners and researchers in interventional cardiology an overview of the past and recent state-of-the-art of ML algorithms and DL architectures, as well as examples of their cardiovascular applications (Table 2). For an all-embracing review of ML and DL approaches applied to cardiology, an avid reader may refer to more exhaustive review papers by Krittanawong et al. (38), Sardar et al. (7), Savakula et al. (82), Bizopoulos and Koutsouris (83), Cuocolo et al. (64), Siegersma et al. (84), Ribeiro et al. (85), and Quer et al. (86).

**Table 2:** Algorithmic overview with prominent examples of implementation in cardiology.

**Type of algorithm**	**Functioning**	**Advantages**	**Drawbacks**	**Implementation**
**SUPERVISED lEARNING**				
Decision trees, random forest, boosting 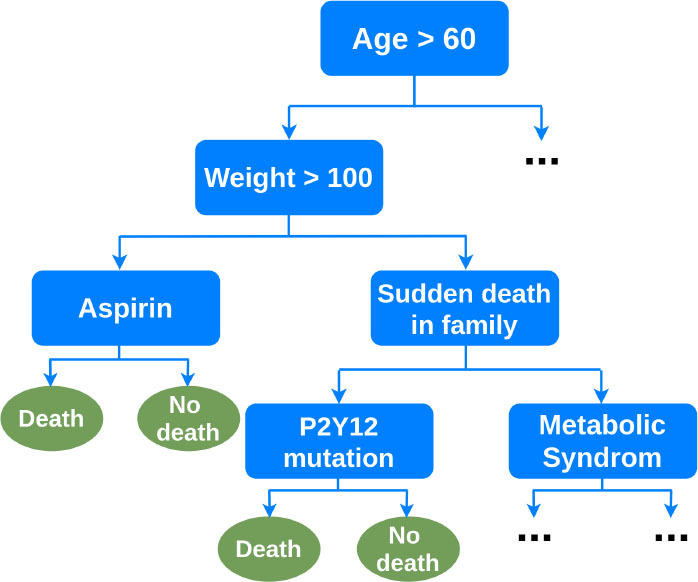	Decision trees are flowchart-type algorithms. Each variable is a condition on which the tree splits into branches, until the outcome “leaf.” Random forest and boosting are it's derivatives.	Interpretability. Integrated feature selection. No preprocessing. Handles non-linear relationships. Requires less data than neural networks.	Computationally expensive. Can overfit or create biased trees in case of unbalanced outcome classes.	Long-term cardiovascular outcomes prediction from clinical, ECG, imaging, biomarker data (15) 5-year mortality prediction from clinical and coronary CT data (48) 30-day readmission after heart failure hospitalization (49, 50) In-hospital mortality prediction after acute myocardial infarction (51) Long-term death or myocardial infarction prediction from coronary CT data (52)
Support vector machine 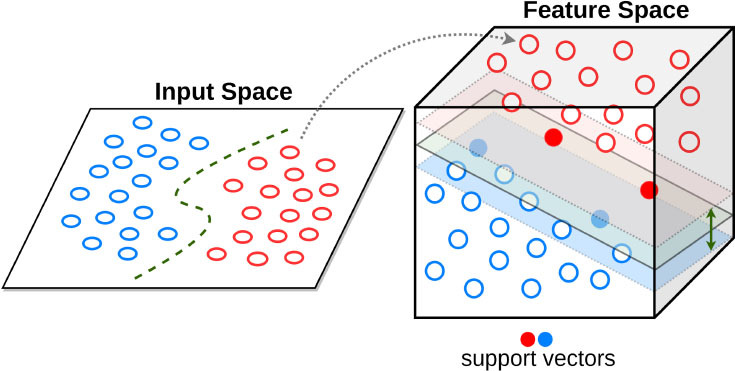	Builds a hyperplane in a high-dimensional space to separate the data into 2 outcome categories with the maximum margin.	Can integrate many sparse features, limits overfitting and is computationally effective	Needs preprocessing. Limited interpretability	Automated echocardiographic assessment of mitral regurgitation (53) Mortality prediction of TAVI outcomes (54)
Regularized regression 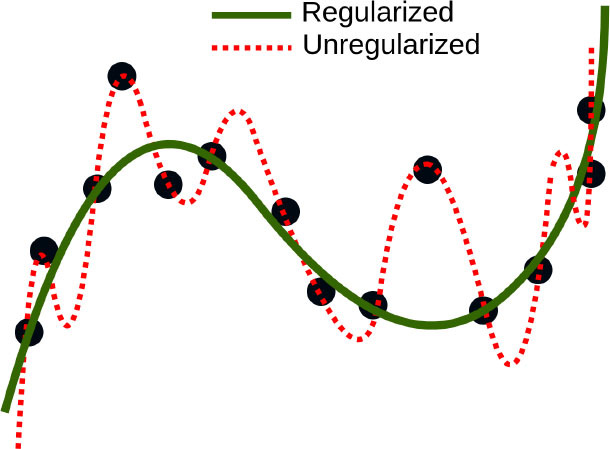	Type of regression where coefficient estimates are constrained by penalty terms (ex: LASSO, ridge)	Familiar interpretations for association of variables to outcomes applied to high-dimensional data	Variable pre-selection is often advisable. Performance stalls for very high-dimensional data	1-year mortality predictors after MitraClip implantation (55) Identification and prediction of adverse clinical outcomes after pediatric cardiac surgery (56)
**UNSUPERVISED LEARNING**				
K-mean clustering 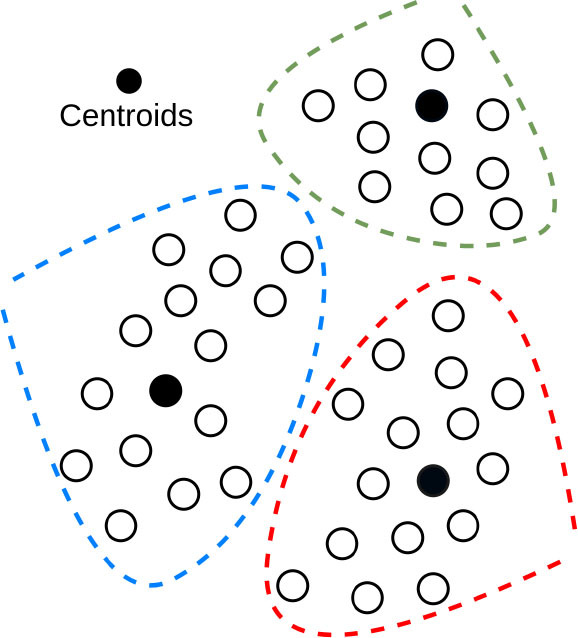	Assigns each data point to a cluster (group; with k the number of groups) based on its distance from the other points	Easy to implementent. Computationally fast.	Number of groups must be known or assigned.	Separate QRS and non-QRS-region in the ECG signal (57)
Principal component analysis 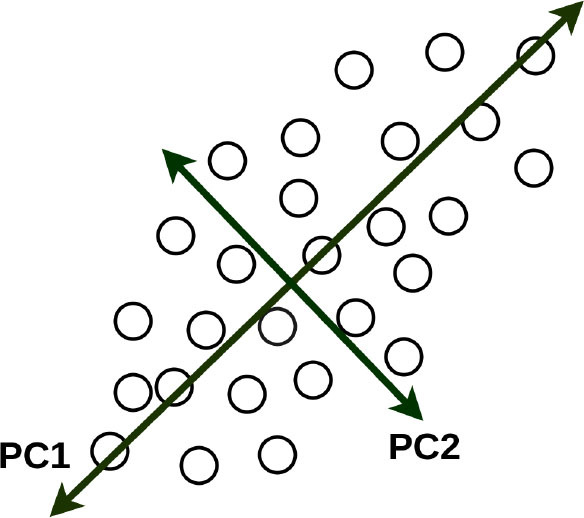	Uses orthogonal transformation to convert possibly correlated variables into a set of linearly uncorrelated principal components.	Can be used for dimensionality reduction.	Only captures linear relationships. Limited interpretability	MACE prediction from clinical and biomarker data representing metabolic syndrome (58) Evaluating 3D aortic shape and hemodynamics (59)
**SHALLOW NEURAL NETWORKS AND DEEP LEARNING (MAINLY USED FOR SUPERVISED LEARNING)**				
Shallow neural networks 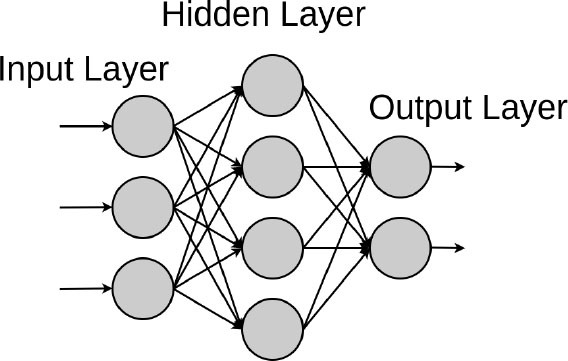	A set of nodes (“neurons”) is arranged in layers connected by edges (weights). The network connects input data to the outcome to predict through a paralleled set of parameterized non-linear transformations.	Can explore non-linear relationships (often encountered in real-life datasets) as well as linear ones. NN can handle heteroskedasticity, have been praised for the generalizability of the trained models, and are computationally effective. Flexible.	Variable pre-selection is often advisable. Needs variable pre-processing.	Diagnosis of coronary artery disease from myocardial perfusion scintigraphy (60)
Deep fully connected neural network 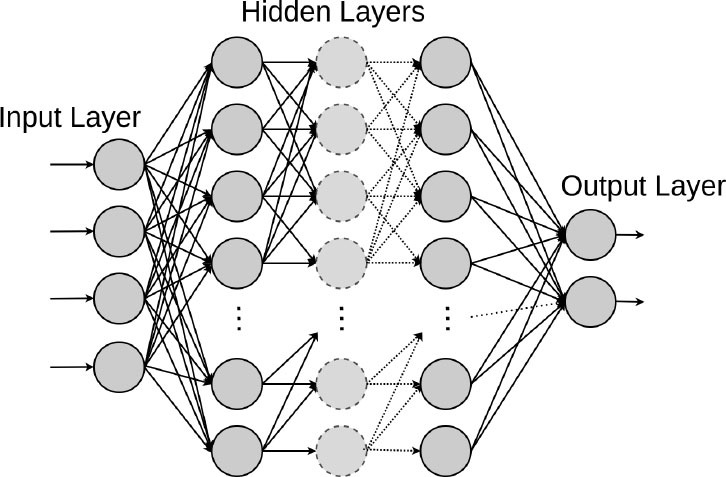	An extension of the shallow NN architecture, but that uses many hidden layers (layers between input and output). Weights and biases of the NN are trained via back-propagation.	Performance increases with the quantity of data. Surpass other machine learning methods for very high-dimensional data. Flexible architecture and basis of CNN, RNN	Requires a high quantity of data. Can easily overfit. Low interpretability Sensible to changes in input data.	Mortality, readmission, LOS and diagnosis prediction from EHR (13) Mid-term mortality prediction from EHR (14) Computation of Fractional Flow Reserve (FFR) from Coronary Computed Tomography (18, 19) Risk stratification for mortality of AMI patients (61)
Convolutional neural network 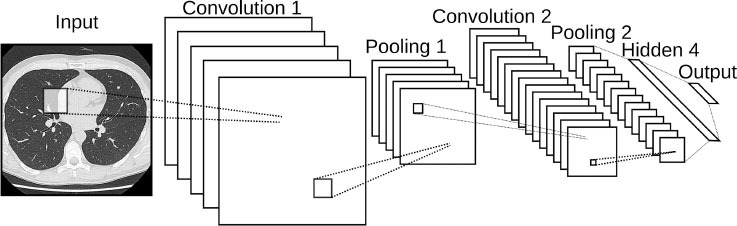	Type of NN which learns multiple levels of feature sets at different levels of abstraction.	One of the most popular deep learning architectures. Flexible. Optimal for image classification.	Requires a high quantity of data. Can easily overfit. Low interpretability	3D aortic valve annulus planimetry in TAVI (62) TTE view identification from images (63) Popular for automated heart chamber segmentation and measurement (64) Early Detection of STEMI (65)
Recurrent neural network 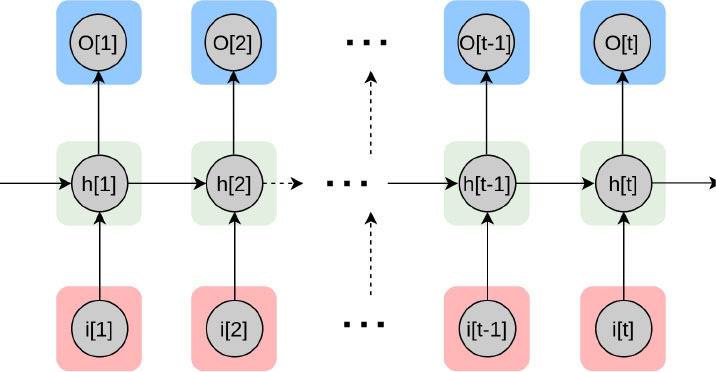	Type of NN which encodes sequential data by capturing context into memory.	Adapted for natural language processing, text or video, genetic sequences or any other temporal data (66–69).	Computationally expensive. Limited quantity of encodable data.	EHR text data extraction for mortality prediction in congenital heart disease (70) Diabetes, high cholesterol, high BP, and sleep apnoea prediction using sensor data (71) Automated selection of myocardial inversion time (72)
**UNSUPERVISED DEEP LEARNING**				
Autoencoder 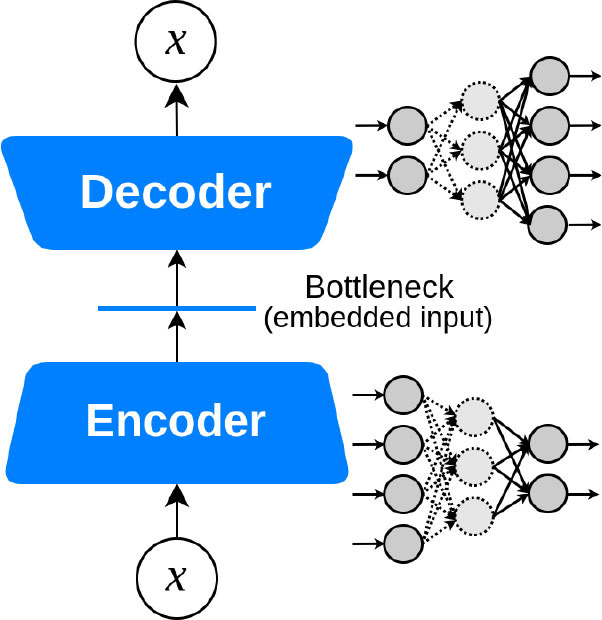	Encodes the most valuable unlabeled inputs into short codes, then uses those to reconstruct the original input as output.	Dimensionality reduction. Optimal for denoising filtering, image segmentation (73).	Low interpretability	MRI-extracted cardiac motion model denoising for survival prediction (74) U-Net for the segmentation of major vessels in X-ray coronary angiography (75)
Deep generative models 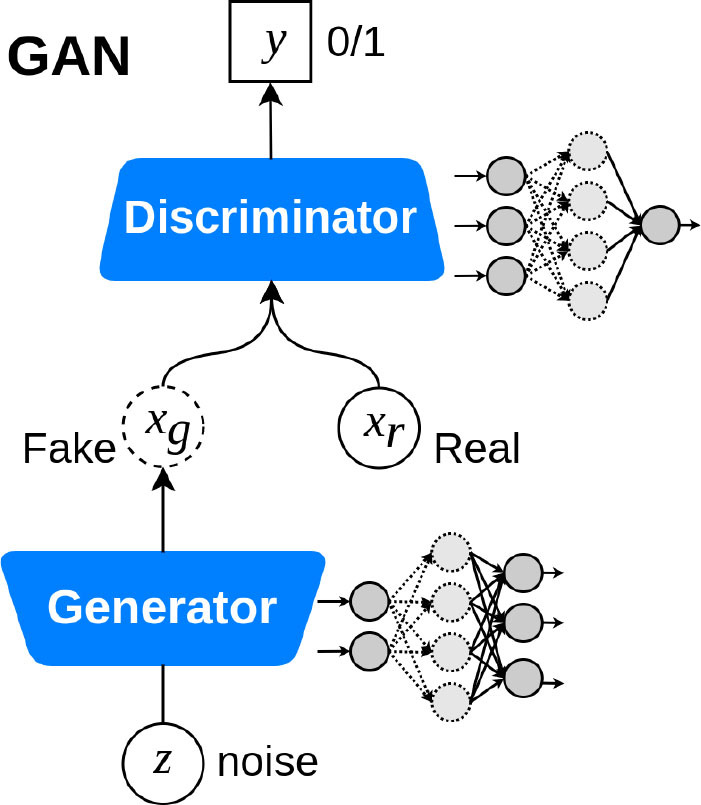	Model a distribution that is as similar as possible to the true data distribution with the help of GANs or VAEs	Data augmentation and preserving data privacy with the help of synthetic data samples. Domain translation and domain adaptation. Content and style matching using adversarial inference (76, 77).	Could be computationally expensive. The models are still in the stage of getting mature for high-fidelity data sample generation. Lack of stability at training time.	Noise reduction in low-dose CT (78) GANs for multiphase coronary CT angiography (25) Synthetic electrocardiogram generation (79)
**REINFORCEMENT LEARNING**				
Deep reinforcement learning 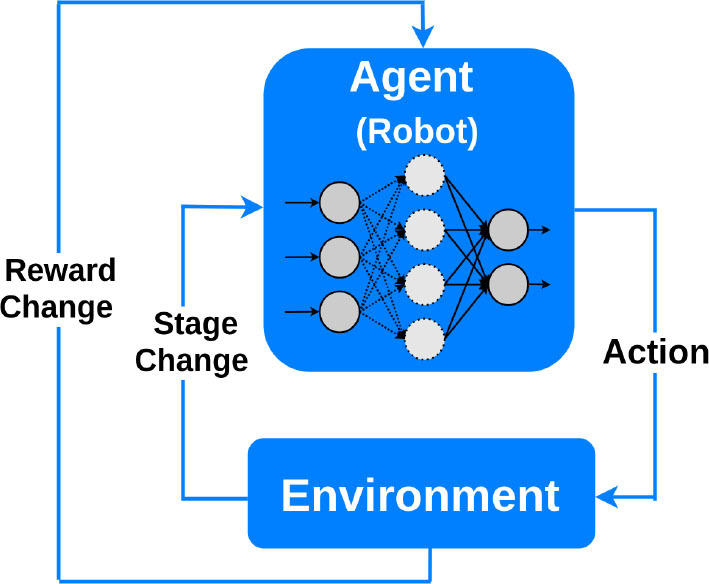	RL learns how to maximize a reward function by exploring the actions available from certain states. A deep RL agent tests an action to see what reward will be returned by the environment in which it acts.	Besides robotic assistance, potential applications include: microbots that can travel through blood vessels to deliver medications; interventional training simulator and tele-intervention (7).	Still in the state of infancy. Complexity and cost. Not preferable to use for solving simple problems. Huge training data demand.	The control of an electrophysiology catheter by robots (32) Robotic-PCI reducing contact with COVID-19 patients undergoing PCI (80, 81)

*AMI, acute myocardial infarction; EHR, electronic healthcare records; LASSO, least absolute shrinkage and selection operator; MACE, major adverse cardiovascular event; NN, neural network; CV, cardiovascular; MRI, magnetic resonance imaging; ECG, electrocardiogram; BP, blood pressure; CT, computed tomography; TAVI, transcatheter aortic valve implantation; PCI, percutaneous coronary intervention; VAE, variational autoencoders; GAN, generative adversarial networks; RL, reinforcement learning; STEMI, ST-segment elevation myocardial infarction*.

Decision Trees (DT) are interpretable supervised learning techniques that can be used for classification or regression (87). They are tree-structured models, where internal nodes represent the features of a dataset, branches represent the learned decision rules, and each leaf node represents the outcome. *Random Forests* (RF) are an ensemble learning method that operates by constructing a multitude of decision trees at training time to correct for overfitting. Other ensembles of trees such as gradient boosted trees, including LogitBoost (88) and XGBoost (89), address the same drawback.

Ambale-Venkatesh et al. tested the ability of RF, to predict several cardiovascular outcomes, including coronary heart and atherosclerotic cardiovascular diseases, in comparison to standard cardiovascular risk scores from clinical, ECG, imaging, and biomarker data (15). They showed the RF technique performed better than established risk scores with high prediction accuracy. Mortazavi et al. and Frizzell et al. worked with clinical data from the index admission, and showed RF methods improved the prediction of readmission after hospitalization for heart failure when compared with logistic regression (LR) and provided the greatest predictive range in observed readmission rates (49, 50). In another application, Motwani et al. investigated the feasibility and accuracy of iterative LogitBoost to predict 5-year all-cause mortality (ACM) in patients undergoing coronary computed tomographic angiography (CCTA) and compared the performance to existing clinical or CCTA metrics (48). They showed combining clinical and CCTA data was found to predict 5-year ACM significantly better than existing clinical or CCTA metrics alone.

Risk stratification and prognosis prediction are critical in identifying high-risk patients and decision making for the treatment of patients with acute myocardial infarction (AMI). Long-existing MI risk scoring systems including TIMI (90), GRACE (91), and ACTION (92) are based on conventional statistical methods, so there is a possibility of a loss of important information. Hence, Khera et al. with the help of the XGBoost model showed an accurate prediction of risk of death following AMI can guide the triage of care services and shared decision-making (51). They studied patients in the Cardiology Chest Pain-MI Registry hospitalized with AMI and discussed contemporary ML may improve risk prediction by identifying complex relationships between predictors and outcomes. The employed registry data included patient demographics, presentation information, pre-hospital vital signs, selected laboratory data from the hospital course, procedures, timing of procedures, and select in-hospital outcomes. Using the XGBoost model also, Rosendael et al. demonstrated an ML-based risk score that utilized standard 16 coronary segment stenosis and composition information derived from detailed CCTA reading had greater prognostic accuracy than current CCTA integrated risk scores (52). They suggested ML-based algorithms can improve the integration of CCTA derived plaque information to improve risk stratification. Similarly, machine learning has been used in small datasets to improve in-stent restenosis over conventional risk scores such as PRESTO-1, PRESTO-2, EVENt and GRACIA-3 (93, 94).

Support Vector Machines (SVM) are popular supervised learning algorithms, which are used for classification and regression problems (95). The goal of the SVM algorithm is to create the best line or decision boundary (or hyperplane) that can segregate high-dimensional space into classes so that we can easily put the new data point in the correct category in the future. SVM chooses the extreme points, i.e., support vectors, that help in creating the hyperplane. Moghaddasi and Nourian have used SVM in the context of Mitral Regurgitation (MR), a common heart disease that does not cause symptoms until its end-stage (53). Early diagnosis of MR is however of crucial importance in the treatment process, and their SVM model with the radial basis function (RBF) kernel function can differentiate between the four groups of Normal, Mild MR, Moderate MR and Severe MR subjects among echocardiography videos. Transcatheter aortic valve implantation (TAVI) has become a commonly applied procedure for high-risk aortic valve stenosis patients. However, for some patients, this procedure does not result in the expected benefits. Lopes et al. demonstrated the accuracy of various traditional ML algorithms, including SVM, RF and XGBoost, in the prediction of TAVI outcomes (54).

Regularized Regression is a type of linear regression where the high-magnitude coefficient estimates are penalized (or regularized) to be small. The regularization methods provide a mean to control the regression coefficients (or weights) in datasets containing a large number of features; this can reduce the variance and decrease the out-of-sample error. Therefore, by appropriate choice of penalizing weights the model prevents overfitting to the training data. Two commonly used types of regularized regression methods are ridge regression (96) and lasso regression (97). Buccheri et al. developed a lasso-penalized Cox-proportional hazard regression model to identify independent predictors of 1-year all-cause mortality, in patients who undergo MitraClip implantation (55). In another study, Wang et al. proposed new variable selection methods for Poisson and naive Bayes regression and used plasma and urine biomarkers to help with early identification and prediction of adverse clinical outcomes after pediatric cardiac surgery (56). They discovered that early postoperative urine biomarkers independently predict prolonged hospital length of stay (LOS).

K-Means Clustering is an unsupervised learning algorithm that groups the unlabeled dataset into different clusters (98). A point is considered to be in a particular cluster if it is closer to that cluster's centroid than any other centroid. K-Means finds the best centroids by alternating between assigning data points to clusters based on the current centroids and choosing centroids based on the current assignment of data points to clusters. Mehta et al. proposed clustering algorithms could be used for the detection of QRS-complexes, the prominent feature of the ECG (57). In their study, the K-Means algorithm was used to separate QRS and non-QRS-region in the ECG signal. The onsets and offsets of the detected QRS-complexes were found well within the tolerance limits.

Principal Component Analysis (PCA) is another popular unsupervised learning algorithm that is used for dimensionality reduction, exploratory data analysis and predictive modeling (99). Agarwal et al. used PCA to derive a continuous measure of metabolic syndrome-based on the multiple interrelated risk factors (58). This metabolic syndrome score was a better predictor of CVD events in multiethnic cohorts than the National Cholesterol Education Program (NCEP) definition, derived predominantly from populations of European ancestry. Quail et al. in their studies evaluated 3D aortic shape and hemodynamics using principal PCA, proposed as an important determinant of adverse hemodynamics following coarctation repair (59). They concluded that shape is not the major determinant of vascular load following coarctation repair, and that caliber is more important than curvature.

Artificial neural networks are ML models that consist of an architecture of intertwined nodes (“neurons”) and edges regrouped in hidden layers connecting the input data and the outputted prediction. Whenever several hidden layers of neurons are used, the model can be described as a deep neural network (DNN), in which millions of connections can be trained in parallel. These algorithms can learn complex non-linear functions to minimize the classification error. We will detail the different DNN model architectures below.

*Shallow Neural Networks* are predecessors of DL. In contrast to deep neural networks, shallow neural networks generally use predefined features, a characteristic that they share with traditional ML algorithms. A study by Guner et al. developed and analyzed an open-source artificial intelligence program built on shallow artificial neural networks that can participate in and support the decision making of nuclear medicine physicians in detecting coronary artery disease (CAD) from myocardial perfusion SPECT (MPS) (60).

Deep Fully Connected Neural Networks (FNN) are networks that consist of multiple perceptrons (i.e., linear binary classifiers) stacked in width and depth. In FNN, every unit in each layer is connected to every unit in the layers immediately before and after. Rajkomar et al. proposed a representation of patients' entire raw EHR records based on the Fast Healthcare Interoperability Resources (FHIR) format (13). They demonstrated that FNN models using this EHR representation were capable of accurately predicting multiple medical events from multiple centers without site-specific data harmonization. Their models achieved high accuracy for tasks such as predicting in-hospital mortality, 30-day unplanned readmission, LOS, and all of a patient's final discharge diagnoses. In the context of palliative care services, Avati et al. proposed an interpretable FNN model trained on the EHR data from previous years, to predict all-cause 3–12 month mortality of patients, as a proxy for patients that could benefit from palliative care (14). Their predictions enabled a palliative care team to take a proactive approach in reaching out to such patients, rather than relying on referrals from treating physicians or conducting time-consuming chart reviews of all patients.

Recently, physics-based models such as computational fluid dynamics (CFD) have shown great promise in being able to non-invasively estimate FFR from patient-specific anatomical information, e.g., obtained from computed tomography scans of the heart and the coronary arteries (100, 101). However, these models have high computational demand, limiting their clinical adoption. Itu et al. developed a FNN for predicting FFR, speeding up physics-based approaches (18). The model is trained on a large database of synthetically generated coronary anatomies, using the physics-based model. They showed that the correlation between ML and physics-based predictions was significant and without systematic bias. Coronary computed tomographic angiography is another reliable modality to detect coronary artery disease. In their study, Coenen et al. showed that on-site CT-fractional flow reserve (CT-FFR) improves the performance of CCTA by correctly reclassifying hemodynamically nonsignificant stenosis (19). Their DNN model performs equally well as computational fluid dynamics-based CT-FFR. Kwon et al. developed an FNN risk stratification model that predicted the in-hospital mortality and 12-month mortality of AMI patients more accurately than the existing risk scores and other ML methods including RF (61). In their model, they used the demographic information and laboratory data of AMI patients as the predictor variables. Such models could be improved by adding more modalities to the input data (e.g., text in EHR and images of CT) as discussed in a study by Myers et al. (102).

Convolutional Neural Networks (CNN), widely used in computer vision, consist of a convolutional and pooling part, where hierarchical feature extraction takes place, and a fully connected part for classification or regression. The models can recognize low-level features, such as edges and corners, and high-level features such as parts of objects thanks to convolutional layers that are much better feature optimizers, while fully connected layers are good classifiers. In TAVI procedures, the sizing of devices is done from ECG-gated CT angiographic image volumes. The most crucial step of the analysis is the determination of the aortic valve annular plane. Theriault-Lauzier et al. developed an expert-level CNN to infer the location and orientation of the aortic valve annular plane (62). Madani et al. investigated the application of CNNs to echocardiography view classification that classified 15 major transthoracic echocardiograms (TTE) views with expert-level quality (63). They used a training set that reflected a wide range of clinical and physiological variations, demonstrating applicability to real-world data. They found that the model used some of the same features in echocardiograms that human experts use to make their decisions. CNNs were also used to fully interpret echocardiograms and diagnose certain diseases with a high level of accuracies such as hypertrophic cardiomyopathy or pulmonary hypertension. These models usually use a single frame to predict the corresponding view or measurement. Recently, video-based AI was used for analyzing a whole echocardiogram video to better predict cardiac function (103). CNNs were also employed for the segmentation of the heart chamber in a work by Cuocolo et al. (64). Segmentation of heart regions in advance can help the subsequent problems in hand. For example, as discussed in their study, segmentation of the epicardium and endocardium from the left ventricle can be important for the assessment of the cardiovascular system function (e.g., hypertrophy vs. normal cases). Most importantly, CNNs can also be used to predict new diseases that were previously not possible. Recently, a CNN was used to derive a digital biomarker that can detect diabetes using a photoplethysmography signal, which is traditionally used for pulse oximetry or for heart rate measurements (104). Such novel digital biomarkers could be derived using data readily available in interventional cardiology, such as coronary angiograms, to predict device failures or certain conditions such as spontaneous coronary artery dissection.

Recurrent Neural Networks (RNN) are ideal for time-series or sequential data. These networks consist of feedback loops, so they can use their internal state to process the input. To estimate prognosis in a large cohort of patients with adult congenital heart disease (ACHD) or pulmonary hypertension, Diller et al. designed an RNNs model that categorized diagnosis and disease stages with high accuracies (70). Ballinger et al., proposed a semi-supervised sequence learning for cardiovascular risk prediction, the DeepHeart model (71). They demonstrated their multi-task RNN model outperforms hand-engineered biomarkers from the medical literature. Working with off-the-shelf wearable heart rate sensors, they suggested that methods such as theirs could help with patient risk stratification based on cardiovascular risk scores derived from popular wearables such as Fitbit, Apple Watch, or Android Wear.

Delayed myocardial enhancement imaging is an essential component of cardiac MRI, which is used widely for the evaluation of myocardial scar and viability (105). The selection of optimal inversion time or null point to suppress the background myocardial signal is required. In their study, Bahrami et al. showed that merging the spatial and temporal characteristics of CNN and LSTM was capable of automated prediction of myocardial inversion time from an inversion-recovery experiment (72). In clinical practice, early ST-segment elevation myocardial infarction (STEMI) detection is of great clinical significance because the very early stages of STEMI are the most vulnerable periods during which most sudden cardiac deaths occur (106); hence, an accurate and efficient warning system based on an ECG can help with patient delay. Zhao et al. proposed a CNN trained on 12-lead ECG that outperforms clinicians in early detection of STEMI (65). They also argue ML-based algorithms have the potential to empower a wide range of physicians to more accurately diagnose STEMI on ECG and reduce the inappropriate activation of catheter labs.

Autoencoders (AE) are neural networks that are trained with the objective to reconstruct the output from the input by encoding useful properties of the data. It usually consists of an encoding part that downsamples the input down to a linear feature and a decoding part that up-samples this representation back to the original dimensions. In a human survival prediction study, Bello et al. used image sequences of the heart acquired using cardiac MRI, to create time-resolved three-dimensional segmentation using a network trained on anatomical shape priors (74). This dense motion model formed the input to a supervised denoising autoencoder, a special AE that randomly turns some input values to zero to prevent overfitting.

U-Net is a modification of the convolutional autoencoders, i.e., encoder-decoder, architecture, first introduced by Ronneberger et al. for medical image segmentation (107). U-Net incorporates additional links between the encoder layers and the decoder layers of the network, resulting in a U-shape structure (107). Although quantitative coronary angiography (QCA) provides morphological information of coronary arteries with objective quantitative measures, considerable training is required to identify the target vessels and understand the tree structure of coronary arteries. Yang et al. proposed a robust method for major vessel segmentation using an adjusted U-Net network (75). Even though the model is evaluated intrinsically with the help of segmentation labels, the same model could be extrinsically used and evaluated by replacing traditional segmentation methods in coronary catheterization for prediction of FFR in intermediate coronary artery lesions (108).

Deep Reinforcement Learning (DRL) uses deep learning and reinforcement learning principles to create efficient algorithms applied to areas such as robotics, natural language processing, computer vision and healthcare. Implementing deep learning architectures with reinforcement learning algorithms is capable of scaling to previously unsolvable problems (25). In a recent work by You et al., a robot was developed to reduce the radiation exposure of personnel during an interventional procedure for arrhythmia (32). Experiments on the control of an electrophysiology catheter by robots were conducted. Using the DRL, they showed that such a robot learned to manipulate a catheter to reach a target in a simulated environment and subsequently control a catheter in an actual environment. Additionally, several studies evaluated the feasibility and technical success of reinforcement learning-based R-PCI for the treatment of CAD in clinical practice when compared with manual PCI (8, 109). As a vivid recent example, to minimize the risk of exposure to severe acute respiratory syndrome coronavirus 2 (SARS-CoV-2) and reduce personal protective equipment needed by the procedural team during the COVID-19 pandemic, studies by Tabaza et al. and Virk et al. showed R-PCI could help to reduce contact with COVID-19 patients undergoing PCI (80, 81).

Deep Generative Models (DGM) are powerful ways of learning any kind of data distribution using unsupervised learning. Since it is not always possible to learn the exact distribution of the data, DGMs try to model a distribution that is as similar as possible to the true data distribution. Two of the most commonly used and relatively efficient approaches are Variational Autoencoders (110) (VAE) and Generative Adversarial Networks (111) (GAN). Considering that advanced image reconstruction from low-dose CT data is needed to improve the diagnostic performance, which is a challenging problem due to its ill-posed nature, Wolterink et al. used a GAN to transform low-dose cardiac CT images into routine-dose CT images (78). In another study, Kang et al. trained a GAN to reduce noise in CCTA images (112). Their proposed unsupervised network learns the image distributions from the routine-dose cardiac phases by eliminating the need to exactly matched low- and routine- dose CT images. A hybrid DL architecture developed by Zhu et al. showed that an LSTM-CNN GAN could generate ECG data with high morphological similarity to real ECG recordings (79). This is of interest, as such an approach could be used to generate a large dataset of ST segment elevation ECGs for training an algorithm that would identified STEMIs due to obstructive CAD vs. non-obstructive disease (pericarditis, for example).

## Implementation of Machine Learning and its Challenges

### AI in the Real World

Since data-driven AI is different from traditional rule-based systems and medical devices it demands adequate control to ensure its safety and effectiveness. Also, because these differences will not be the same for the full range of systems, it is important to identify what aspects of AI are of concern (113). The safety and effectiveness of medical devices entering the market today are governed by regulations and private-sector consensus standards. Whereas, in most cases, they were developed alongside current technologies and are based on an extensive, shared understanding of how and how well they work. With an emergent technology like AI, real-world experience is limited, which can hinder regulators and practitioners' ability to fully assess its effectiveness. Similarly, a lack of real-world experience with AI limits the understanding of its associated risks. AI-related risks are harder to quantify and mitigate as there may be unforeseeable and unpredictable hazards arising from the unique nature or function of AI. This is particularly important in raw health data that generally lack maintenance and validation and raise important interoperability problems (113, 114). AI may became untrustworthy also because data was not representative or not fit for the task to which it was applied. The availability of data is essential as a source of information for training AI systems, but it is also a source of noise, especially when data quality is poor, labeling is inconsistent, or sampling is biased. Iterative preprocessing of data must be done before it is considered to be of adequate quality for downstream ML tasks, such that quality management of data is understood as an important issue by AI practitioners. Recently, there is a push toward a more data-centric approach to ML to increase accuracy based on improving the datasets (115), in contrast to the widespread model-centric approach that focuses on changing the model to improve performance. Improving the quality of a dataset does not necessarily mean increasing dataset size, it can be achieved by fixing incorrect labels, adding examples that represent edge cases, or apply data augmentation.

Furthermore, the nature of the application either rule-based, data-driven locked (i.e., non-adaptive through time), or data-driven while continuously learning (i.e., life-long learning), as well as the context of application which can be informative or provide decision support with or without a human in the loop play major roles (113). Given that AI or data has the ability to change over time, the processes of verification and validation cannot be a onetime premarket activity, but instead must continue over the life cycle of an AI system from the initial design and clinical substantiation, across its post market use, until decommissioning. Such life cycle consists of data quality assurance, pre-market risk management and assurance of effectiveness, pre-specification and algorithm change, and real-word performance monitoring. Continual assurance of the AI-based device's safety and performance across its life cycle will help regulators, clinicians, and patients gain trust in data-driven AI. To this end, a recent initiative by a group of medical device regulators from several countries, including Food and Drug Administration (FDA) from USA, has established International Medical Device Regulators Forum (IMDRF) to harmonize the regulatory requirements for medical products under a notion named Software as a Medical Device (SaMD) (116). The FDA's Center for Devices and Radiological Health (CDRH) is also considering a total product life-cycle-based regulatory framework for these technologies that would allow for modifications to be made from real-world learning and adaptation, while ensuring that the safety and effectiveness of the software as a medical device are maintained (117). As further advancements are made in AI technology, regulators will need to consider additional approaches for addressing the safety and effectiveness of AI in healthcare, including how international standards and other best practices are currently used to support the regulation of medical software, along with differences and gaps that will need to be addressed for AI solutions. One key aspect will be the need to generate real-world clinical evidence for AI systems throughout their life cycles, and the potential for additional clinical evidence to support adaptive systems. Next to AI systems themselves, regulators must also consider that there are ethical, social and political challenges comprising issues regarding trust, liability, privacy and risk (118). These complexities of applications of AI require further reflection, proof of their medical utility, economic valuing, and development of interdisciplinary strategies for their wider application (119).

Last but not least, the capacity of complex decision-making in interventional cardiology or in performing a procedure independently would be very challenging for current AI and ML algorithms. Understandably, by considering the speed of progress and development, AI technologies could not completely replace human interventional cardiologists in the foreseeable future. It can be easily anticipated, however, that AI will widely assist rather than replace the human operator in the catheterization laboratory. Hence, the reception and integration of AI in a specialty which needs quick decision-making by the operator should be discussed and practiced prior to the actual deployment of innovations brought by ML and DL models.

### Domain Expertise

Beyond AI algorithm development, several additional issues should be tackled before implementing AI into clinical practice (86). First, domain-experts, such as cardiologists should collaborate with data scientists an AI engineers in order to jointly develop AI algorithms that is as bias free as possible, respects the regulatory framework for development and addresses a clinically relevant need (86, 120). By addressing accurate and reliable implementation of ML and DL algorithms in cardiology, the Proposed Requirements for Cardiovascular Imaging-Related Machine Learning Evaluation (PRIME) checklist provided by Sengupta et al. lays down seven items to be reported for reducing algorithmic errors and biases aiming to standardize reporting on model design, data, selection, assessment, evaluation, replicability, and limitations (121). Next, clinical trials need to be conducted to demonstrate that such algorithms are positively influencing morbidity, mortality or healthcare delivery. The SPIRIT-AI and CONSORT-AI working groups have put forward guidelines for clinical trials for interventions involving AI and represent a framework by which to conduct and report such trials (122, 123). It is crucial to perform extensive external validation on multiple datasets to demonstrate the algorithm's robustness. Finally, once the algorithm is found to positively influence healthcare, further real-world quality control must be conducted to assure that the algorithm is providing accurate predictions and that its performance does not deteriorate over time. This could be done by routinely collecting feedback on the predictions from the domain-experts. Further, continuous learning of AI Algorithms, post-commercialization, to improve predictions on reported errors and to adapt to new data, remains an area of active research.

Large electronic healthcare databases (LEHD) are being built from electronic health records and already used by several countries to implement AI in healthcare (124, 125). Some prominent examples of such databases are the UK Biobank (UK), Million Veterans Initiative (USA), NIH precision medicine initiative (USA), large Scandinavian national registries in Denmark, Sweden, and Norway. If dispersed big data are to disrupt current research models then there is a need for searchable catalogs of data, metadata, feasibility counts (and ideally sample data) and access arrangements. The creation of public, standards-driven metadata and data portals can assist researchers in locating the right dataset for their research question and obtaining up to date details on data availability and accessibility. Moreover, contemporary LEHD often contain multi-omics data (transcriptomics, genomics, proteomics, metabolomics, microbiomics, radiomics) intended for deep phenotyping of patients (126, 127). However, the size does not always ensure the precision of the model built, nor that the intent of improving care for all people is met. What is more, further progress in automation of data harvesting and inter-database harmonization (e.g., EHR and national statistical organisms which record vital status) would facilitate the construction of high-quality high-dimensional databases (128).

### Underspecification

As stated above, the quality of the training samples provided to an ML algorithm is of central importance in data-driven AI. This is because ML models often exhibit unexpectedly poor behavior when they are deployed in real-world domains due to underspecification (129). An ML or DL pipeline could be underspecified when it returns many predictors (e.g., several predictive models with distinct and dissimilar weights) with equivalently strong held-out performance in the training domain yet these models perform significantly different when generalized, therefore questioning the credibility of the predictors in practice. For many medical applications, a key challenge is the robustness of the ML model under the distribution shift of data in the deployment domain, and as a result, several studies confirm the need for explicitly testing and monitoring ML models in settings that accurately represent the deployment domain (122, 123, 130). In addition, heterogeneity in the representation of different ethnicities, gender inequalities, socioeconomic status, geography in datasets could generate biased estimations and automate inequalities (131). Therefore, to address underspecification, next to improving the training and testing process, and also considering multiple ML models as alternatives at deployment time, limiting model complexity as well as designing stress tests to probe stratified performance evaluations, shifted evaluations, and contrastive evaluations should be considered (129).

**Figure 2:**
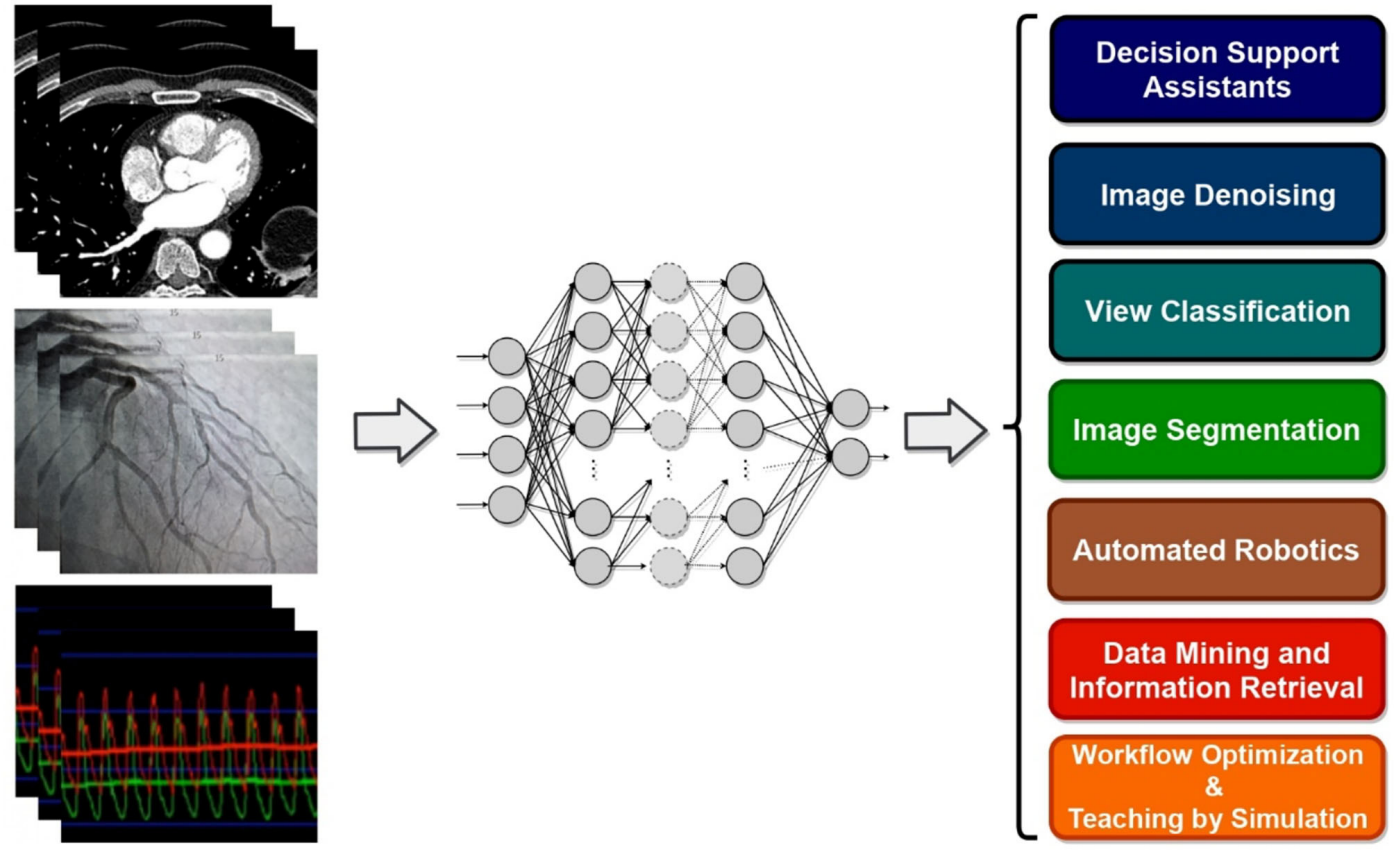
Domains of implementation of machine learning tools to cardiology.

Despite their performance, expecting to achieve perfect prediction with DL models is probably vain. The chaos theory states that even with a deterministic (non-random) process, even simple non-linear systems cannot be precisely predicted into the distant future (11, 132). Conventional statistical approaches often use a standardized stepwise approach. After univariate feature analysis, a model is selected and uses cohorts with manually entered structured databases. This differs from the machine learning approach which tends to avoid model selection and uses “fuzzier” emerging sources of data that are more prone to contain some quantity of bias (11, 70). Without appropriate oversight, ML models can easily overfit in noisy datasets, impairing their capacity to generalize to new data due to over-interpretation of noise (Figure 1). This is particularly true when the number of examples (patients) are limited compared to the number of variables measured for each patient or when the outcome of interest is of rare occurrence, which is often the case in some present-day medical applications. Besides, building predictive models is inherently based on past events, and the future will not necessarily resemble the past, nor will they necessarily perform well on a population different from the one represented in the training cohort (11). Numerous teams have successfully applied DL algorithms to yield high-performance predictive models through the mining of EHR with the idea of assisting doctors through decision-support algorithms by combining all the available information, irrespective of their time of occurrence (11, 13, 14, 35). However, for decision-support algorithms to be implemented in clinical practice, we would expect them to be accurate and pertinent at the time the decision is taken, without assuming to know everything in advance (Figure 2) as developed by Diller et al. to guide therapy in adult congenital heart disease (70).

### Overfitting and Interpretability

Despite ML models being theoretically superior to usual statistical models in terms of predictive power (15, 133), their practical use must also be rigorous along with its reporting and reviewing (134). Furthermore, the computationally demanding DL algorithms also require efficient programming libraries (such as PyTorch and TensorFlow), and specific hardware (such as Graphics or Tensor Processing Unit instead of the usual Central Processing Unit). Additionally, all ML and DL models may suffer from overfitting if data is limited and/or algorithms are complex. Indeed, in clinical studies, DL provided similar results to statistical models (e.g., logistic regression) (38). Transfer learning, data augmentation with the help of deep generative models, as well as integration of different data sources can be solutions to overfitting problems, but in some cases the curse of dimensionality will prevent some types of analyses on small datasets. Future studies may integrate DL with statistical classification. Furthermore, doctors and patients would also need to understand the exact reasons that led to a medical decision. However, evaluating ML model decisions can be a very difficult task. Once a model is trained, it requires additional approaches to understand the reason behind a particular prediction to a set of data inputs (135). In particular, the numerous intertwined relationships captured by the layers of a DNN are only partially understood, leading to being frequently labeled as “black boxes,” and the observed trade-off between accuracy and interpretability of machine learning models (136). Explaining single predictions or the entire model behavior of DNNs is important to correct their malfunctions, bias, and susceptibility to slight modifications of analyzed data (137). Interpretability may be enabled by capsule based networks or strategies that systematically censor inputs to define those that most affect classification. Meta-analyses of several DL algorithms applied to the same data may increase confidence in results. A number of techniques may enable “model-agnostic” metrics for interpretability of complex models (138). Marblestone et al. (139) hypothesized analogies between DL and human cognitive functioning, proposing that integrating heterogeneous “cost functions” over time may simplify learning. Thus, speculatively, insights into human cognition may ultimately provide insights to interpret DL models. Encouragingly, much research is ongoing aiming at improving our understanding of ML and DL models (140).

### Missing Data

Similar to statistical tasks, the performance of DL can be highly sensitive to missing data as well. Missing data is a common problem in routine medical records, hence, measures of data management and pre-processing should be addressed in line with the extra complexity they impose on the robustness ML (141, 142). Decisions on how to treat missing data can be made by evaluating if the presence or absence of specific elements correlates with desired outcomes or predictors. Those data that are correlated are “non-ignorable,” those that are not correlated may be “ignorable” (i.e., no relationship to any variables) (143). Additionally, instead of omitting patients with missing data, it is ideal to impute missing data points to obtain more patients for training process and ML analysis. Using k-nearest neighbor (k-NN) to fill in missing values of a data point with the closest known ones, or simply relying on mean or most frequent values of variables to fill in missing positions are standard approaches. Another popular method for imputing missing values is called “multiple imputation using chained equations” (MICE) (144). MICE statistically measures the uncertainty of the missing values and is able to impute different variable types (i.e., continuous, unordered and ordered categorical, etc.) that may reside in the medical records while each variable is imputed by its own model. Another frequently used imputation technique in mixed data is the “factor analysis of mixed data” (FAMD) algorithm (145). FAMD is a principal component method which balances the influence of all the variables that are continuous and categorical in the construction phase of the dimensions. MICE and FAMD, however, are computationally intensive making them suboptimal for pre-processing steps in DL models. Hence, designing strategies, developing imputation algorithms, and their suitable evaluation for realistic settings of medical domain are of great importance. While still an active area of research, many studies have already shown desirable imputation results obtained by autoencoders models such as denoising autoencoders (146, 147).

## Ethical Issues: Patient Data Misuse Must Be Avoided

A current worrying tendency to exploit patient data for financial purposes must be acknowledged, discussed, and acted upon. A Dutch startup CathSuite aims to automatically extract patient information from various catheterization laboratory report sources and hospitals, and store it in a standardized form, notably on mobile phones. Among their intended purposes of data use is research, but also monetization through contracts with insurance companies (148). It is appropriate that patient data is extracted with their consent for research purposes that will aim at improving healthcare. However, it is hardly conceivable that patient data could be exploited for private company financial gains, at the detriment of the patient, by sharing their data with insurance companies or other private actors that could use it against patients.

Data privacy has been the subject of the European General Data Protection Regulation (149). A legislative context is lacking for the specific context of patient data protection, although protecting patient data could be even more important than the data of healthy individuals. De-identification should not be seen as inviolable protection since the power of ML algorithms could allow the data extraction and storing of the path to be reversed and traced back to the patient. Reports of EHR data breaches are not infrequent (150). The Hippocratic Oath states “Whatever I see or hear in the lives of my patients, whether in connection with my professional practice or not, which ought not to be spoken of outside, I will keep secret, as considering all such things to be private.” Information and Technology (IT) professionals, private companies wishing to exploit patient data, let alone insurance companies, do not abide by the medical secret, the main guardian of the patient-physician relationship of care. More than ever, physicians must protect their patients' data, verify that its use is intended at improving care, and guard against monetization at the detriment of patients. Regulators and international medical associations must address the gap in the guidelines of patient data exploitation and provide limitations of possible applications to research. Companies and researchers must be held responsible for the data they are entrusted with, their use of it and the tools they create to exploit it, including data misuse.

Furthermore, letting private industry companies shaping the future of AI is not the only path toward progress in medicine through technology. An industrial profit-maximizing approach is likely to diverge from public interest (23). Independent quality research is important. Hospitals should employ data scientists and IT professionals under hospital authority to ensure patient data protection and appropriate exploitation directed at improving healthcare. An upgrade of cyber protection of hospital informatics systems storing EHRs should be considered.

### Final Comments

Once the hype of AI is passed, a backlash against this very promising field of research remains possible. Reticence from patients toward the use of their data, and physicians' reluctance to the use of technology as an intermediate between them and patients could fuel discontent. Patient misuse must not be tolerated. Recent progress in the field of AI interpretability suggests that this setback can be overcome (136) and the focus should be on developing approaches that are human-interpretable, to allow reliable strategies to be deployed to assist clinicians in their medical practice. Several trials are ongoing to develop algorithms predicting procedural success, in-hospital mortality, and 1-year mortality after transcatheter aortic valve replacement. This is of big interest, especially in the era of expanding indications to lower risk and younger population, to help heart teams in the decision-making process and in the selection of optimal candidates and devices.

## Author Contributions

WB, AP, and JH: substantial conception, drafting of the article for important intellectual content, and revising the article for final approval of the submitted paper. RA: drafting of the article for important intellectual content and revising the article for final approval of the submitted paper. PO and NP: drafting of the article for important intellectual content. SL, RC, RI, and TM: revising the article for final approval of the submitted paper. All authors contributed to the article and approved the submitted version.

## Funding

RA received support from the Fonds de la recherche en santé du Québec (FRSQ) (grant 274831). JH is a FRSQ Junior 1 Research Scholar (252997) and is funded by IVADO (PRF-2019-3378524797) and Montreal Heart Institute Foundation (FICM-1132).

## Conflict of Interest

RA owns NVIDIA, a company making graphic cards for artificial intelligence analyses, stocks. RA received speaker fees from Novartis Inc. and has a patent pending (R31-07141: METHOD AND SYSTEM FOR ASSESSMENT OF VENTRICULAR EJECTION FRACTION). JH received speaker honoraria from District 3 Innovation Centre and DalCor Pharmaceuticals. The remaining authors declare that the research was conducted in the absence of any commercial or financial relationships that could be construed as a potential conflict of interest.

## Publisher's Note

All claims expressed in this article are solely those of the authors and do not necessarily represent those of their affiliated organizations, or those of the publisher, the editors and the reviewers. Any product that may be evaluated in this article, or claim that may be made by its manufacturer, is not guaranteed or endorsed by the publisher.
